# The effect of professional-led guideline workshops on clinical practice for the management of patent ductus arteriosus in preterm neonates in Japan: a controlled before-and-after study

**DOI:** 10.1186/s13012-015-0258-5

**Published:** 2015-05-08

**Authors:** Tetsuya Isayama, Xiang Y Ye, Hironobu Tokumasu, Hiroo Chiba, Hideko Mitsuhashi, Sadequa Shahrook, Satoshi Kusuda, Masanori Fujimura, Katsuaki Toyoshima, Rintaro Mori, the Neonatal Research Network of Japan

**Affiliations:** Department of Newborn and Developmental Paediatrics, Sunnybrook Health Sciences Centre, Toronto, ON Canada; Maternal-Infant Care Research Center, Mount Sinai Hospital, Toronto, ON Canada; Department of Consultation, Kurashiki Clinical Research Institute, Kurashiki, Okayama, Japan; Division of Pediatrics, Sendai City Hospital, Sendai, Miyagi, Japan; Maternal and Perinatal Center, Tokyo Women’s Medical University, Tokyo, Japan; Department of Health Policy, National Center for Child Health and Development, 2-10-1, Okura, Setagaya-ku, Tokyo, 157-8535 Japan; Osaka Medical Center and Research Institute for Maternal and Child Health, Izumi, Osaka, Japan; Department of Neonatology, Kanagawa Children’s Medical Center, Yokohama, Kanagawa, Japan

**Keywords:** Pediatrics, Neonatology, Preterm infants, Clinical practice guideline, Patent ductus arteriosus, Premature infants, Mortality, Morbidity, Information dissemination, Translational medical research

## Abstract

**Background:**

Clinical guidelines assist physicians to make decisions about suitable healthcare. We conducted a controlled before-and-after study to investigate the impact of professional-led guideline workshops for patent ductus arteriosus (PDA) management on physicians’ clinical practices, discharge mortality, and associated morbid conditions among preterm neonates.

**Methods:**

We recruited physicians practicing at two neonatal intensive care units (NICUs) in Japan and used the data of all neonates weighing less than or equal to 1,500 g admitted to 90 NICUs (2 intervention NICUs and 88 control NICUs) in the Neonatal Research Network of Japan from April 2008 to March 2010. We held 1-day workshops for physicians on PDA clinical practice guidelines at the two intervention NICUs. Physicians’ skills assessed by confidence rating (CR) scores and the Sheffield Peer Review Assessment Tool (SPRAT) were compared between pre- and post-workshop month at the intervention NICUs using Wilcoxon signed-rank tests. Neonatal discharge mortality and morbidity were compared between pre- and post-workshop year at both the intervention and control NICUs using multivariable regression analyses adjusting for potential confounders.

**Results:**

Fifteen physicians were included in the study. Physicians’ CR scores (2.14 vs. 2.47, *p* = 0.02) and SPRAT (4.14 vs. 4.50, *p* = 0.05) in PDA management improved after the workshops. The analyses of neonatal outcomes included 294 and 6,234 neonates in the intervention and control NICUs, respectively. Neonates’ discharge mortality declined sharply at the intervention NICUs (from 15/146 to 5/148, relative risk reduction −0.67; adjusted odds ratio 0.30, 95% confidence interval 0.10 to 0.89) during the post-workshop period. The mortality reduction was much greater than that in the control NICUs (from 207/3,322 to 147/2,912, relative risk reduction −0.19; adjusted odds ratio 0.75, 95% confidence interval 0.59 to 0.95), although the difference between the intervention and control NICUs were not statistically significant.

**Conclusions:**

Overall, physicians’ confidence in PDA management improved after attending guideline workshops. Face-to-face workshops by guideline developers can be a useful strategy to improve physicians’ PDA management skills and, thereby, might reduce PDA-associated mortality in preterm neonates.

**Electronic supplementary material:**

The online version of this article (doi:10.1186/s13012-015-0258-5) contains supplementary material, which is available to authorized users.

## Background

About 70% of preterm neonates at <28 weeks of gestational age are widely affected by patent ductus arteriosus (PDA) [1], and about one-third of very low birth weight (VLBW) neonates are symptomatic [2]. PDA is a cardiac condition that might be associated with infant mortality [3,4] and morbidity such as bronchopulmonary dysplasia (BPD) [5,6], intraventricular hemorrhage (IVH) [7], and necrotizing enterocolitis (NEC) [8,9]. No causality has been established between PDA and these morbidities [10,11] nor any evidence favoring specific treatment that is likely to decrease the associated mortality or severe morbidities; however, closure of PDA has been successful [12]. Although numerous PDA therapies were universally accepted, in due course, they were proven ineffective, harmful, and periodically catastrophic [13]. As a result, PDA management has been constrained by complex uncertainties in diagnosis, allocation of clinical priority, treatment identification, and preferred management modality [14] that have led to gaps between evidence and routine practice. Furthermore, different practices exist within and between countries [15-17]. For instance, in Japan, wide variations are reported in indomethacin use, enteral feeding adjustment, fluid management, and ventilation strategies, although diagnosis criteria between institutions do not vary [18]. These inconsistencies have resulted in confusion and uncertainty in the medical community and raise questions about treatment ethics. Under these uncertainties, standard PDA treatment should follow ‘risks versus benefits’ rationale quantified by robust evidence tailored to the conditions of individual neonates [10,11].

Translating research into daily practice and changing physicians’ behavior could be a daunting challenge for various reasons, e.g., the massive amount of generated evidence [19] and different levels of healthcare system delivery (e.g. professional and patient-level) might inhibit practice change [19]. In such predicaments, clinical guidelines might be useful to uphold treatment effectiveness and to discourage ineffective treatment exercises [20]. Among various educational strategies to endorse professional practice and execution of research findings, targeted planning e.g., outreach visits by experts, interactive workshops, and multifaceted program have shown consistent usefulness, whereas the evidence was inconsistent for the effectiveness of other strategies such as passive dissemination of educational materials, involvement of opinion leaders, audit and feedback, and instructive educational sessions [19,21]. Also, the effectiveness of the approaches may vary by types of target professional performances, societal or cultural factors, and differences in healthcare systems [21,22]. In neonatology, evidence on effective educational methods is lacking except for that on neonatal resuscitation [23,24]. An absence of effective methods to implement guidelines therefore could either hinder the improvement of treatment quality or lead to hazardous clinical outcomes, especially in a sensitive population such as neonates.

Therefore, in this study, we hypothesized that workshops on PDA management guidelines by guideline developers will have a positive effect on physicians’ change in practice and will thereby reduce the associated adverse outcomes in preterm neonates, including mortality. We held face-to-face workshops at two tertiary-level neonatal intensive care units (NICUs) in Japan, which were used as intervention sites, and sought to evaluate their impact on physicians’ routine practices and clinical outcomes in premature neonates with PDA before and after the workshops.

## Methods

### Study design, setting, and participants

This was a controlled before-and-after study conducted in tertiary-level NICUs in Japan. The study included 15 physicians from two intervention NICUs—Kurashiki Central Hospital (KCH) and Osaka Medical Center and Research Institute for Maternal and Child Health (OMCRI-MCH)—and all VLBW neonates weighing ≤1500 g admitted to the Neonatal Research Network of Japan (NRNJ) from April 2008 to March 2010. The two intervention NICUs were selected by convenience and by the study investigators. The included intervention sites were eligible as they were the central NICUs providing tertiary-level care in individual region. The NICUs offer a range of crucial healthcare services such as obstetrics and cardiac or general pediatric surgeries. At the point of this study, the NICUs managed extremely preterm neonates (22–25 weeks of gestational age), including neonates with surgical requirement. The other NICUs (*N* = 88) under the NRNJ network served as controls in this study.

### Intervention

**Figure 1:**
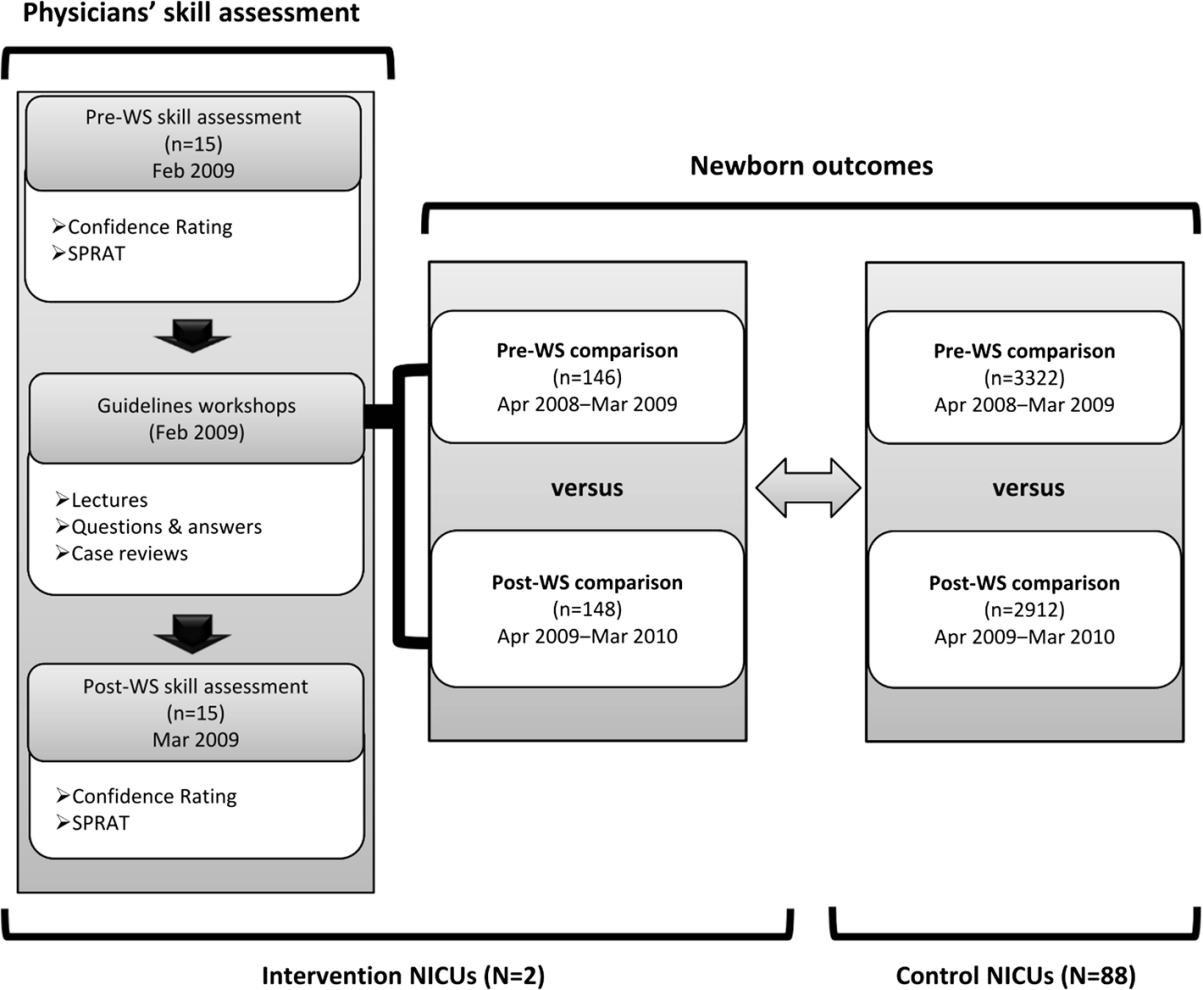
Flow diagram of the study. Legend: WS: workshop.

We held 1-day workshops on the clinical practice guideline for PDA management in preterm neonates [25,26] in February 2009 to assess the workshop impact on practice change at KCH and OMCRI-MCH. The guideline used in the workshop was developed according to the international standards for assessing the quality of practice guidelines (AGREE) [27] and published originally in Japanese [25,26]. All the recommendations made in the Japanese guideline version were translated in English and available as an Additional file 1. Two neonatologists from the guideline development group organized and led the workshops, which consisted of a 2-hour lecture on the guidelines, a question-and-answer session, and case reviews (Figure 1). The first half of the lecture focused on the guideline development process, including question selection, formulating recommendations from evidence mapping, and consensus building through the refined Delphi process [28]. The remainder of the lecture presented the final recommendations of the guidelines and their rationale. In the case reviews, the lecturers and participants discussed several PDA cases (their clinical courses and problems related to PDA). The case selection was particularly driven by the usual difficulty surrounding PDA management (e.g., poor response to the treatment and delayed case detection) and related adverse outcomes (respiratory deterioration, bowel problems, renal failure, etc.).

It has been suggested that physicians’ practice change could be hindered by various factors embedded deeply in individual contexts; therefore, it is likely that the change will vary from one setting to another [19]. Furthermore, evidence effectiveness on multifaceted educational interventions, educational outreach visits, interactive workshops, and several others are found consistent [21]. We therefore designed a multifaceted intervention consisting of educational material (guidelines), educational outreach visits by content experts, face-to-face interactions (question-and-answer sessions [Q&A] and case reviews), and local consensus developing process (case reviews). The national guideline and content experts helped overcome the potential barriers possibly aroused from a lack of motivation, leadership, knowledge, and skills to adopt the current evidence and clinical practice change [19]. The content experts were selected mainly based on their proficient expertise in evidence-based medicine and PDA management and experiences in organizing similar educational training in Japan; however, they did not receive any targeted training for this study. Case reviews helped to develop a consensus for the implication of the guidelines into the local context.

Participating physicians did not receive any information about their outcome evaluation at baseline i.e., management skill measures from confidence rating (CR) and the Sheffield Peer Review Assessment Tool (SPRAT) and neonates’ clinical outcomes. There were no interventions in the control NICUs.

### Outcome measures

#### Pre- and post-assessment of physicians’ PDA management skills in the intervention NICUs

We assessed the impact of the workshops on physicians’ practice change 1 month before and after using a structured questionnaire on PDA management in the intervention NICUs (KCH and OMCRI-MCH) only. We evaluated confidence levels using CR scores [29] and clinical skill assessment using SPRAT [30]. CR is a validated method to assess trainee physicians’ self-reported confidence in a scale ranges from 1 to 4: not confident (CR 1), satisfactory but lack confidence (CR 2), confident in some cases (CR 3), and fully confident in most of the cases (CR 4) [29]. The workshop physicians self-rated the CR for PDA diagnosis, prophylaxis, and treatment management (Additional file 2). SPRAT was used to peer review the participants and consists of 24 questions covering good clinical care, good medical practice, teaching and training, physician-patient relationships, and working relationships with colleagues, plus an overall measurement [30]. Using a 6-grade performance scale, in which 1 is the lowest and 6 is the highest, reviewers assigned the trainees as appropriate. The questions in SPRAT have been described previously [30]. One senior neonatologist from each of the intervention sites (was not a workshop participant) rated the participants’ performance based on how well they complied with the guideline recommendations for PDA management (Additional file 1). The evaluation time of the physicians’ practicing skill was selected arbitrary based on our assumption that the change would require at least a month to detect an intervention effect. We hypothesized that without any educational training, physicians’ confidence and skill in daily practice would not change *in a month*. Assessments for CR and SPRAT were not approached in the control NICUs.

#### Neonatal data and endpoints in the intervention and control NICUs

We reviewed data on VLBW neonates weighing ≤1500 g admitted at the intervention NICUs from 1 April 2008 to 31 March 2010 and compared these data with the VLBW neonates at the control NICUs of NRNJ. The NRNJ is a large multicenter network, of which most tertiary-level NICUs are members. We excluded neonates with known congenital anomalies or moribund infants (no resuscitative care administration was decided at birth). Data abstractors collected data on VLBW neonates admitted to all NICUs using a standard network database operation manual. Primary outcomes were physicians’ PDA management skills and neonatal mortality at discharge. Secondary outcomes included significant PDA or PDA based on echocardiography; indomethacin use to treat PDA; PDA ligation; respiratory distress syndrome (RDS); BPD defined as oxygen use at 36 weeks corrected gestational age with oxygen use on 28th day after birth; severe IVH of grade 3 or 4 [31]; periventricular leukomalacia (PVL); stage 2 NEC or higher based on Bell’s criteria [32]; retinopathy of prematurity (ROP) of grade 3 (middle, late) or above, as per Japanese ROP grading guidelines; late onset sepsis; air leak syndrome; and persistent pulmonary hypertension of the newborn (PPHN).

### Statistical analyses

The sample sizes (number of physicians and neonates) were derived based upon practicality and feasibility of available resources at the time this study was conducted. As this was a pilot study, no existing relevant study from Japan was available to justify our sample size calculation.

To assess the impact of the workshop, we compared physicians’ skills (average CR and SPRAT scores in each of their assessed domains) between pre- and post-workshop period using Wilcoxon signed-rank test. The maternal and neonatal characteristics were compared between the pre- and post-workshop years (April 2008 to March 2009 and April 2009 to March 2010, respectively) using Chi-square tests, Fisher’s exact tests, and *t*-tests. Multivariable logistic regression analyses compared neonatal outcomes between pre- and post-workshop periods, adjusting for potential confounders i.e., gender, gestational age, Apgar score at 5 minutes, C-section, antenatal steroid use, maternal hypertension, chorioamnionitis, outborn, and the interaction term between the periods (pre- vs. post-workshop) and sites (intervention vs. control NICUs). A significant interaction term between the periods and sites indicates a significant difference in the intervention impact between the intervention and control NICUs. The ORs with 95% confidence interval (CI) determination in the regression model were separately reported for both the intervention and control sites. We also conducted difference-in-differences (DID) analysis for the binary outcomes accounting for aggregate time effect [33,34]. We addressed missing data using complete case analyses and excluded observations with missing predictor variables. The data management and statistical analyses were performed using PASW statistics 18.0 (SPSS Inc., Hong Kong) and SAS 9.3 (SAS Institute, Inc., Cary, NC). A significant level of 2-sided *p* value <0.05 was used.

### Ethical approval

This study was approved by the ethical committees of the Kurashiki Central Hospital and Osaka Medical Center and Research Institute for Maternal and Child Health. Written informed consent for data collection and usage were obtained from all physicians included in the study. Data collection from the NRNJ database was approved by the internal review board at the Tokyo Women’s Medical University. Written informed consent for data collection and usage at the NRNJ were obtained from the parents or guardians of all included neonates.

## Results

### Physicians’ PDA management skills

Fifteen physicians participated in the workshops and questionnaire (100% response rate; no missing data). Following the workshops, this study found a significant increase in overall CR (pre- vs. post-workshop: 2.14 vs. 2.47, *p* = 0.02) and a borderline increase in overall SPRAT score (4.14 vs. 4.50, *p* = 0.05) (Table 1). Some domain scores in SPRAT e.g., good clinical care and teaching and training had marked post-workshop increases (*p* = 0.03 and 0.04).

**Table 1:** **Pre- and post-workshop comparisons of physicians’ PDA management skills**

**Confidence rating**	**Pre-WS mean (SD)**	**Post-WS mean (SD)**	***p*** **value**
Prophylactic indomethacin	2.13 (0.99)	2.53 (1.13)	0.14
Treatment of symptomatic PDA	2.20 (0.86)	2.60 (0.99)	0.06
Decision of PDA ligation	1.87 (0.83)	2.40 (0.99)	0.02
Monitoring during indomethacin administration (e.g., signs and complications)	2.60 (0.91)	2.73 (0.80)	0.41
Other PDA treatments (e.g., transfusion, diuretics)	2.00 (0.76)	2.33 (0.82)	0.03
PDA treatments in chronic phase	1.87 (0.99)	2.20 (0.77)	0.21
Fluid management during PDA treatments	2.20 (0.94)	2.47 (0.83)	0.16
Respiratory management during PDA treatments	2.13 (0.74)	2.47 (0.83)	0.06
Nutrition during PDA treatments	2.27 (0.88)	2.47 (0.99)	0.32
Overall	2.14 (0.76)	2.47 (0.80)	0.02
Sheffield Peer Review Assessment Tool			
Good clinical care	4.11 (0.56)	4.49 (0.76)	0.03
Maintain good medical practice	3.95 (0.52)	4.36 (0.77)	0.03
Teaching and training	4.10 (0.99)	4.43 (0.92)	0.04
Relationship with patients	4.67 (0.77)	4.89 (0.97)	0.08
Working relationships with colleagues	4.13 (0.36)	4.43 (0.73)	0.09
Overall	4.14 (0.42)	4.50 (0.71)	0.05

Wilcoxon signed-rank test was used, and *p* value <0.05 was considered statistically significant. There were no missing data. Pre-WS: before the workshops; Post-WS: after the workshops; 95% CI: confidence interval; confidence rating score range: 1 to 4; Sheffield Peer Review Assessment Tool score range: 1 to 6

### Maternal and neonatal characteristics

The analyses of neonates’ data included 294 (pre-workshop: 146; post-workshop: 148) and 6,234 (pre-workshop: 3,322; post-workshop: 2,912) neonates in the two intervention NICUs and the other 88 control NICUs, respectively. We observed a significant post-workshop difference in chorioamnionitis and antenatal steroids use in both the intervention and control NICUs as well as a significant increase in maternal hypertension, premature rupture of the membrane, and C-section in the control NICUs (Table 2).

**Table 2:** **Pre- and post-workshop comparisons of maternal and neonatal characteristics**

	**Intervention NICUs (** ***N*** **= 2)**				**Control NICUs (** ***N*** **= 88)**			
Variables	Pre-WS (*n* = 146)	Post-WS (*n* = 148)	*p* value	MD or OR (95% CI)	Pre-WS (*n* = 3,322)	Post-WS (*n* = 2,912)	*p* value	MD or OR (95% CI)
Maternal age (years)	31.2 ± 5.1	31.6 ± 5.4	0.56	0.43 (−0.78, 1.64)	31.2 ± 5.2	31.4 ± 5.3	0.37	0.12 (−0.15, 0.39)
Missing	1	0			166	47		
Primipara	121 (82.9)	116 (78.4)	0.32	0.75 (0.42, 1.34)	2,836 (85.4)	2,514 (86.3)	0.27	1.08 (0.94, 1.25)
Multiple pregnancy	42 (28.8)	40 (27.0)	0.73	0.92 (0.55,1.53)	834 (25.1)	681 (23.4)	0.11	0.91 (0.81, 1.02)
Maternal hypertension	22 (15.1)	25 (16.9)	0.67	1.15 (0.61, 2.14)	625/3,320 (18.8)	589/2,815 (20.9)	0.04	1.14 (1.01, 1.29)
Maternal diabetes	3 (2.1)	3 (2.0)	0.98	0.99 (0.20, 4.97)	44/3,320 (1.3)	52/2,749 (1.9)	0.08	1.44 (0.96, 2.15)
PROM	51 (34.9)	47 (31.8)	0.56	0.87 (0.53, 1.41)	896/3,322 (26.9)	866/2,888 (30.0)	<0.01	1.16 (1.04, 1.29)
Chorioamnionitis	19/146 (13.0)	46/146 (31.5)	<0.01	3.08 (1.70, 5.58)	494/3,271 (15.1)	467/2,652 (17.6)	<0.01	1.20 (1.05, 1.38)
Antenatal steroids use	80/146 (54.8)	97/147 (66.0)	0.05	1.6 (0.998, 2.57)	1,446/3,320 (43.6)	1,474/2,827 (52.1)	<0.01	1.41 (1.28, 1.56)
Gestational age (weeks)	28.2 ± 3.6	28.4 ± 3.2	0.67	0.19 (−0.58, 0.98)	28.4 ± 3.2	28.3 ± 3.2	0.82	−0.02 (−0.14, 0.18)
Birth weight (grams)	1,001.2 ± 323.9	991.7 ± 322.3	0.8	−9.53 (−83.6, 64.5)	1,027.2 ± 303.6	1,032.7 ± 301.7	0.47	5.53 (−9.53, 20.59)
Birth length (cm)	34.8 ± 4.1	34.4 ± 4.5	0.45	−0.38 (−1.39, 0.62)	35.3 ± 4.0	35.3 ± 4.2	0.85	0.02 (−0.19, 0.23)
Missing	4	1			232	158		
Head circumference (cm)	25.2 ± 2.7	25.4 ± 3.3	0.5	0.24 (−0.46, 0.95)	25.6 ± 2.8	25.6 ± 2.9	0.9	−0.01 (−0.16, 0.14)
Missing	8	1			296	188		
Gender (male)	80 (54.8)	72 (48.7)	0.29	0.78 (0.49, 1.24)	1,670/3,322 (50.3)	1,505/2,911 (51.7)	0.26	1.06 (0.96, 1.17)
Outborn	9 (6.2)	5 (3.4)	0.26	0.53 (0.17, 1.63)	245 (7.4)	225 (7.7)	0.6	1.05 (0.87, 1.27)
Vertex presentation	95/146 (65.1)	100/144 (69.4)	0.42	1.22 (0.75, 1.99)	2,258/3,309 (68.2)	1,859/2,658 (70.0)	0.15	1.08 (0.97, 1.21)
Cesarean section	110 (75.3)	105 (70.9)	0.39	0.80 (0.48, 1.34)	2,502 (75.3)	2,268 (77.9)	0.02	1.15 (1.03, 1.30)
Apgar score < 7 at 5 min	36/145 (24.8)	36/148 (24.3)	0.92	0.97 (0.57, 1.66)	801/3,219 (24.9)	682/2,863 (23.8)	0.33	0.94 (0.84, 1.06)

Chi-square, Fisher’s exact test, or *t*-test was used as appropriate. *p* value <0.05 was considered statistically significant. All the estimates were rounded. Binary outcome variables with missing data are indicated by the denominators showing the numbers of assessed infants. *MD* mean difference, *OR* odds ratio, *CI* confidence intervals. MD or OR estimates correspond as appropriate. Unless otherwise stated, values represent number of neonates with their percentages in the parentheses. *NICUs* neonatal intensive care units, *PROM* premature rupture of the membrane; Pre-WS: before the workshops; Post-WS: after the workshops.

### Neonatal clinical outcomes

**Figure 2:**
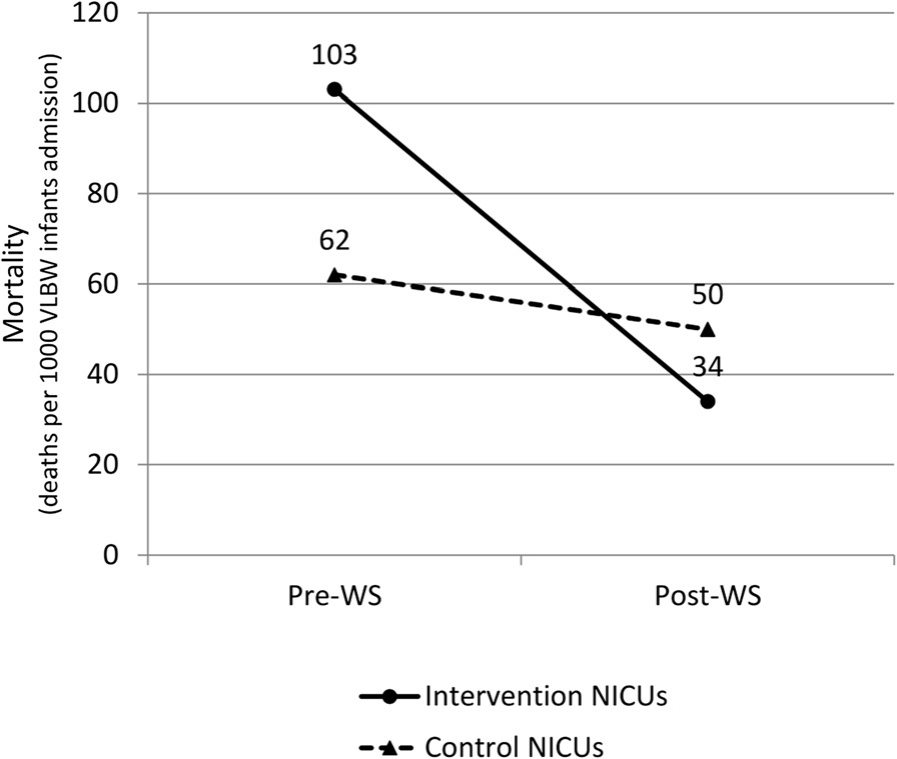
Mortality in intervention NICUs and control NICUs before and after the workshop. Legend: VLBW: very low birth weight, WS: workshop, NICUs: neonatal intensive care units.

The odds of neonates’ discharge mortality improved significantly (from 15/146 to 5/148, relative risk reduction [RRR] −0.67; adjusted odds ratio [AOR] 0.30, 95% CI 0.1 to 0.89) during the post-workshop period, after adjusting for potential covariates (Table 3). There was also a significant mortality decline at the control sites (from 207/3,322 to 147/2,912, RRR −0.19; AOR 0.75, 95% CI 0.59 to 0.95) during the same period, and it was greater in the intervention sites than in the controls (Figure 2), although no statistical significance was observed. (*p* = 0.075). Similarly, the difference in reduction of mortality rate (−5.71%, 95% CI −11.6 to 0.12) between intervention and control sites went in favor of the intervention sites, although no statistical significance was observed. Additionally, we observed slight increases in the odds of a few variables in the control sites only during the post-workshop year. No significant differences for PDA, indomethacin administration, and PDA ligation were detected in the intervention and control sites.

**Table 3:** **Pre- and post-workshop comparisons of neonatal clinical outcomes**

	**Intervention NICUs (** ***N*** **= 2)**				**Control NICUs (** ***N*** **= 88)**				**Intervention vs. control group difference**
Outcomes	Pre-WS *n* = 146 (%)	Post-WS *n* = 148 (%)	Unadjusted OR (95% CI)	Adjusted OR (95% CI)	Pre-WS *n* = 3,322 (%)	Post-WS *n* = 2,912 (%)	Unadjusted OR (95% CI)	Adjusted OR (95% CI)	DID in post- vs. pre-WS (95% CI)**
Mortality	15 (10.3)	5 (3.4)	0.31 (0.11, 0.86)*	0.30 (0.1, 0.89)*	207 (6.2)	147 (5.1)	0.80 (0.64, 0.99)*	0.75 (0.59, 0.95)*	−5.71 (−11.6, 0.12)
RDS	80 (54.8)	90 (60.8)	1.28 (0.81,2.04)	1.55 (0.90, 2.68)	1,893/3,322 (57.0)	1,682/,2884 (58.3)	1.06 (0.96, 1.17)	1.05 (0.93, 1.18)	4.68 (−6.86, 16.2)
BPD	25/141 (17.7)	36/147 (24.5)	1.51 (0.85, 2.67)	1.66 (0.90, 3.07)	490/3,282 (14.9)	504/2,858 (17.6)	1.22 (1.07,1.40)*	1.23 (1.06, 1.44)*	4.05 (−5.51, 13.6)
Severe IVH	6 (4.1)	8 (5.4)	1.33 (0.45, 3.94)	1.38 (0.45, 4.27)	145/3,322 (4.4)	126/2,882 (4.4)	1.00 (0.78, 1.28)	1.0 (0.77, 1.30)	1.29 (−3.68, 6.26)
PVL	3/146 (2.1)	6/143 (4.2)	2.09 (0.51, 8.51)	2.09 (0.51, 8.51)	112/3,318 (3.4)	91/2,864 (3.2)	0.94 (0.71, 1.24)	1.0 (0.75, 1.34)	2.34 (−1.77, 6.45)
ROP	14/139 (10.1)	17/140 (12.1)	1.23 (0.58, 2.61)	1.50 (0.66, 3.41)	289/2,429 (11.9)	360/2,512 (14.3)	1.23 (1.05, 1.46)*	1.33 (1.11, 1.60)*	0.36 (−7.97, 7.24)
NEC	3 (2.1)	3 (2.0)	0.98 (0.19, 4.97)	1.11 (0.22, 5.72)	55/3,322 (1.7)	53/2,889 (1.8)	1.11 (0.76, 1.62)	1.01 (0.68, 1.51)	−0.21 (−3.5, 3.09)
Early sepsis	3 (2.1)	2 (1.4)	0.65 (0.11, 3.97)	0.65 (0.11, 3.97)	88 (2.6)	92 (3.2)	1.20 (0.89, 1.61)	1.27 (0.94,1.73)	−1.21 (−4.29, 1.86)
Late sepsis	7 (4.8)	6 (4.1)	0.84 (0.27, 2.56)	0.93 (0.30, 2.91)	168/3,313 (5.1)	172/2,880 (6.0)	1.19 (0.95, 1.48)	1.17 (0.93, 1.48)	−1.64 (−6.48, 3.20)
PDA	47 (32.2)	45 (30.4)	0.92 (0.56, 1.51)	0.97 (0.57, 1.65)	1,286/3,322 (38.7)	1,109/2,884 (38.5)	0.99 (0.89, 1.10)	0.95 (0.85, 1.07)	−1.53 (−12.4, 9.35)
Indomethacin	43 (29.5)	44 (29.7)	1.01 (0.61, 1.67)	1.01 (0.64, 1.86)	1,229 (37.0)	1,054 (36.2)	0.97 (0.87, 1.07)	0.93 (0.83, 1.05)	1.08 (−9.63, 11.8)
PDA ligation	13 (8.9)	7 (4.7)	0.51 (0.20, 1.31)	0.46 (0.17, 1.23)	208/3,322 (6.3)	185/2,865 (6.5)	1.03 (0.84, 1.27)	0.96 (0.77, 1.20)	−4.37 (−10.3, 1.51)
Air leak	7 (4.8)	4 (2.7)	0.55 (0.16, 1.93)	0.66 (0.17, 2.22)	88/3,322 (2.7)	93/2,880 (3.2)	1.23 (0.91, 1.65)	1.22 (0.90, 1.66)	−2.67 (−7.09, 1.75)
PPHN	11 (7.5)	15 (10.1)	1.38 (0.61, 3.12)	1.33 (0.57, 3.17)	132/3,322 (4.0)	152/2,872 (5.3)	1.35 (1.06, 1.71)*	1.39 (1.09,1.79)*	1.28 (−5.28, 7.85)

Variables with missing data are indicated by the denominators showing the numbers of the assessed neonates. Covariates included in the multiple logistic regression models were gender, gestational age, Apgar score < 7 at 5 minutes, cesarean section, antenatal steroids use, maternal hypertension, chorioamnionitis, and outborn.

Pre-WS: before the workshops; Post-WS: after the workshops. *OR* odds ratio, 95% *CI* 95% confidence interval, *RDS* respiratory distress syndrome, *BPD* bronchopulmonary dysplasia, *IVH* intraventricular hemorrhage, *PVL* periventricular leukomalacia, *ROP* retinopathy of prematurity, *NEC* necrotizing enterocolitis, *PDA* patent ductus arteriosus, *PPHN* persistent pulmonary hypertension.

*Statistically significant at the 5% probability level; **DID: The difference in differences of neonatal outcomes (percentage points) = differences (%) between the post- and pre-WS for the intervention NICUs − differences (%) between the post- and pre-WS for the control NICUs, estimated using a probit model.

## Discussion

To our knowledge, this is the first study to examine the effects of professional-led guidelines implementation workshops on physicians’ management skills of PDA and neonatal mortality and morbidities. We found that face-to-face outreach visits by guideline developers were effective in improving physicians’ confidence and skills and may reduce mortality in preterm neonates.

Positive workshop impact in improving professionals’ practices and health outcomes is supported by an earlier Cochrane review [22]. Our findings add further support to the likelihood that changing physicians’ behavior is achievable [35], even in a highly controversial field like PDA [10-12,14,36]. Administered care could potentially be unnecessary or harmful, if the new evidence uptake is ineffectively practiced [37,38]. This is especially important in Japan where preterm neonates are frequently monitored by functional echocardiography before receiving tailored care. Additionally, augmenting new evidence and a daily recommended uptake [39] might be challenging for clinicians [40]. Guideline translation could also be challenging due to its content and dissemination strategy [19]. Therefore, it is crucial that these issues are effectively addressed in guideline implementation to influence physicians’ routine practice [21]. Although no single strategy has proven superior [19,41], our study provides support to the effectiveness of a multifaceted educational intervention consisting of educational material (clinical guideline), expert outreach visits, and face-to-face interactive meetings including local consensus developing process (question-and-answer sessions and case reviews) to reduce the gap between research and practice [21]. Additionally, the effects of any targeted interventions for behavior change are context-dependent [21]. A unique work environment surrounding Japanese physicians such as extremely long work hours [42] might make the workshop more effective because it is often difficult for them to have enough time to update their clinical knowledge and skill without well-organized educational sessions.

Similar to the intervention sites, mortality reduction in the control NICUs was also significant and was consistent with previous findings that reported an ongoing, marked improvement among VLBW neonates’ mortality in Japan [43,44]. Although the reduction was greater in the intervention sites compared to the controls, it was not statistically significant; therefore, we could not differentiate the positive workshop impact from the background declining trend of neonatal mortality. However, this should not diminish the importance of our findings because the guidelines were not intended to reduce PDA and its treatment rate but to reduce the risks of associated clinical deterioration and adverse outcomes by providing tailored care for a specific neonatal condition. Though insignificant, the PDA ligation rate in the intervention sites dropped during the post-workshop period, which may indicate an improvement in physicians’ PDA management skills.

The strengths of our study include the use of a quasi-experimental design, a national registry database, and the employment of validated tools and standard guideline development methods [30]. This comprehensive process might collectively influence physicians to comply with recommendations and encourage better practice adherence [19,45]. Our study has illustrated the potential impact of the guidelines on neonatal clinical outcomes, for which evidence is scarce [23,24]. Furthermore, our quasi-experimental design provides important complementary information when relevant trials are limited [46]. Our study has several limitations. First, our sample size included a small number of workshop participants, warranting larger sample sizes in future studies. Second, physicians’ assessment of CR was self-reported, which increases risk of recall bias. Although the SPRAT was validated in a previous study including general pediatricians at tertiary and secondary hospitals in the UK as a multisource peer review tool [30], its reliability in this study setting (neonatologists and pediatricians in Japanese NICUs) as a single-source peer review was uncertain [30]. Third, due to a lack of information, we could not investigate several management modalities, e.g., adoption of prophylactic with indomethacin and fluid and nutrition. Fourth, because of the study design, we could not exclude the possibility that improved mortality rates were caused by other factors. Fifth, although we conducted a 1-day workshop due to the practicality and feasibility of the available resources, more frequent workshops than a day-long workshop in this study could have a large effect [47]. Finally, the external validity of this study is limited by the setting (tertiary hospitals in Japan); therefore, the findings must be interpreted with caution due to wide variations in PDA management between institutions and countries.

## Conclusions

Face-to-face workshops by guideline developers can be a useful strategy to improve physicians’ confidence and skills for PDA management and thereby might reduce PDA-associated mortality in preterm neonates especially in Japan. Our findings have added important knowledge to this area at a time when evidence on effective guideline implementation strategy in everyday practice is lacking, particularly in Japan. We propose an enhanced uptake of PDA management guidelines by increasing the number of professional-led workshops in Japan to stimulate guideline development and disseminate effective implementation strategies for better practice adherence. Future studies could provide further evidence by employing large multicenter cluster randomized controlled trials to evaluate the effectiveness of organization- and patient-oriented interventions [41].

## Additional files

Additional file 1:
**Evidence- and consensus-based clinical practice guideline for treatment of patent ductus arteriosus (PDA) in preterm infants (English version).** This file is an English translation of 33 recommendations in the ‘Evidence and Consensus Based Clinical Practice Guideline for Treatment of Patent Ductus Arteriosus of Preterm Infants’ that was originally published in Japanese. Additional file 2:
**Evaluation of physicians’ confidence for PDA diagnosis, prophylaxis, and treatment management.** This file is a questionnaire on physicians’ confidence for PDA diagnosis, prophylaxis, and treatment. Additional file 3:
**Site investigators of the Neonatal Research Network of Japan.** This file is a full list of the names of site investigators of the Neonatal Research Networks of Japan.

## Abbreviations

AOR: Adjusted odds ratio
BPD: Bronchopulmonary dysplasia
CI: Confidence interval
CR: Confidence rating scores
DID: Difference in difference
IVH: Intraventricular hemorrhage
KCH: Kurashiki Central Hospital
MD: Mean difference
NEC: Necrotizing enterocolitis
NICUs: Neonatal intensive care units
NRNJ: Neonatal Research Network of Japan
OR: Odds ratio
OMCRI-MCH: Osaka Medical Center and Research Institute for Maternal and Child Health
PDA: Patent ductus arteriosus
PVL: Periventricular leukomalacia
PPHN: Persistent pulmonary hypertension of the newborn
RRR: Relative risk reduction
RDS: Respiratory distress syndrome
ROP: Retinopathy of prematurity
SPRAT: Sheffield Peer Review Assessment Tool
VLBW: Very low birth weight
WS: Workshop

## References

[1] Clyman RI (2000). Ibuprofen and patent ductus arteriosus. N Engl J Med.

[2] The Investigators of the Vermont-Oxford Trials Network Database Project (1993). The Vermont-Oxford Trials Network: very low birth weight outcomes for 1990. Investigators of the Vermont-Oxford Trials Network Database Project. Pediatrics.

[3] Noori S, McCoy M, Friedlich P, Bright B, Gottipati V, Seri I (2009). Failure of ductus arteriosus closure is associated with increased mortality in preterm infants. Pediatrics.

[4] Brooks JM, Travadi JN, Patole SK, Doherty DA, Simmer K (2005). Is surgical ligation of patent ductus arteriosus necessary? The Western Australian experience of conservative management. Arch Dis Child Fetal Neonatal Ed.

[5] Rojas MA, Gonzalez A, Bancalari E, Claure N, Poole C, Silva-Neto G (1995). Changing trends in the epidemiology and pathogenesis of neonatal chronic lung disease. J Pediatr.

[6] Marshall DD, Kotelchuck M, Young TE, Bose CL, Kruyer L, O'Shea TM (1999). Risk factors for chronic lung disease in the surfactant era: a North Carolina population-based study of very low birth weight infants. North Carolina Neonatologists Association Pediatrics.

[7] Evans N, Kluckow M (1996). Early ductal shunting and intraventricular haemorrhage in ventilated preterm infants. Arch Dis Child Fetal Neonatal Ed.

[8] Cassady G, Crouse DT, Kirklin JW, Strange MJ, Joiner CH, Godoy G (1989). A randomized, controlled trial of very early prophylactic ligation of the ductus arteriosus in babies who weighed 1000 g or less at birth. N Engl J Med.

[9] Dollberg S, Lusky A, Reichman B (2005). Patent ductus arteriosus, indomethacin and necrotizing enterocolitis in very low birth weight infants: a population-based study. J Pediatr Gastroenterol Nutr.

[10] Bose CL, Laughon MM (2007). Patent ductus arteriosus: lack of evidence for common treatments. Arch Dis Child Fetal Neonatal Ed.

[11] Clyman RI, Chorne N (2007). Patent ductus arteriosus: evidence for and against treatment. J Pediatr.

[12] Gournay V, Roze JC, Kuster A, Daoud P, Cambonie G, Hascoet JM (2004). Prophylactic ibuprofen versus placebo in very premature infants: a randomised, double-blind, placebo-controlled trial. Lancet.

[13] Silverman WA (1980). Retrolental fibroplasia: a modern parable.

[14] McNamara PJ, Sehgal A (2007). Towards rational management of the patent ductus arteriosus: the need for disease staging. Arch Dis Child Fetal Neonatal Ed.

[15] Amin SB, Handley C, Carter-Pokras O (2007). Indomethacin use for the management of patent ductus arteriosus in preterms: a web-based survey of practice attitudes among neonatal fellowship program directors in the United States. Pediatr Cardiol.

[16] Hoellering AB, Cooke L (2009). The management of patent ductus arteriosus in Australia and New Zealand. J Paediatr Child Health.

[17] Guimaraes H, Rocha G, Tome T, Anatolitou F, Sarafidis K, Fanos V (2009). Non-steroid anti-inflammatory drugs in the treatment of patent ductus arteriosus in European newborns. J Matern Fetal Neonatal Med.

[18] Toyoshima K, Masumoto K, Aoyanagi Y, Yota H. Standardization of medicine: diagnosis and treatment of PDA. Nationwide questionnaire survey on clinical practice of patent ductus arteriosus. Journal of Japanese Society of Premature and Newborn Medicine (Japanese). 2008;20:452.

[19] Grol R, Grimshaw J (2003). From best evidence to best practice: effective implementation of change in patients' care. Lancet.

[20] NHMRC. How to put the evidence into practice: implementation and dissemination strategies. 2000.

[21] Bero LA, Grilli R, Grimshaw JM, Harvey E, Oxman AD, Thomson MA (1998). Closing the gap between research and practice: an overview of systematic reviews of interventions to promote the implementation of research findings. BMJ.

[22] O'Brien MA, Rogers S, Jamtvedt G, Oxman AD, Odgaard-Jensen J, Kristoffersen DT (2007). Educational outreach visits: effects on professional practice and health care outcomes. Cochrane Database Syst Rev.

[23] Patel D, Piotrowski ZH, Nelson MR, Sabich R (2001). Effect of a statewide neonatal resuscitation training program on Apgar scores among high-risk neonates in Illinois. Pediatrics.

[24] Perlman JM, Wyllie J, Kattwinkel J, Atkins DL, Chameides L, Goldsmith JP (2010). Part 11: Neonatal resuscitation: 2010 International Consensus on Cardiopulmonary Resuscitation and Emergency Cardiovascular Care Science with Treatment Recommendations. Circulation.

[25] Japanese Preterm PDA (J-Prep) Guideline Development Team (2010). Evidence and consensus based clinical practice guideline for treatment of patent ductus arteriosus of preterm infants. J Japan Soc Premature and Newborn Med.

[26] Japanese Preterm PDA Guideline Group (J-PreP Guideline Group) (2010). Clinical practice guideline for the management of patent ductus arteriosus in preterm or low birth weight infants. Japan Society for Premature and Newborn Medicine.

[27] The AGREE Collaboration (2003). Development and validation of an international appraisal instrument for assessing the quality of clinical practice guidelines: the AGREE project. Qual Saf Health Care.

[28] van der Linde H, Hofstad CJ, van Limbeek J, Postema K, Geertzen JH (2005). Use of the Delphi technique for developing national clinical guidelines for prescription of lower-limb prostheses. J Rehabil Res Dev.

[29] George JT, Warriner DA, Anthony J, Rozario KS, Xavier S, Jude EB (2008). Training tomorrow's doctors in diabetes: self-reported confidence levels, practice and perceived training needs of post-graduate trainee doctors in the UK. A multi-centre survey. BMC Med Educ.

[30] Archer JC, Norcini J, Davies HA (2005). Use of SPRAT for peer review of paediatricians in training. BMJ.

[31] Papile LA, Burstein J, Burstein R, Koffler H (1978). Incidence and evolution of subependymal and intraventricular hemorrhage: a study of infants with birth weights less than 1,500 gm. J Pediatr.

[32] Bell MJ, Ternberg JL, Feigin RD, Keating JP, Marshall R, Barton L (1978). Neonatal necrotizing enterocolitis. Therapeutic decisions based upon clinical staging. Ann Surg.

[33] Dimick JB, Ryan AM (2014). Methods for evaluating changes in health care policy: the difference-in-differences approach. JAMA.

[34] Lui K, Abdel-Latif ME, Allgood CL, Bajuk B, Oei J, Berry A, Henderson-Smart D (2006). Improved outcomes of extremely premature outborn infants: effects of strategic changes in perinatal and retrieval services. Pediatrics.

[35] Grimshaw J, Eccles M, Tetroe J (2004). Implementing clinical guidelines: current evidence and future implications. J Contin Educ Health Prof.

[36] Knight DB, Laughon MM (2008). Evidence for active closure of patent ductus arteriosus in very preterm infants. J Pediatr.

[37] Schuster MA, McGlynn EA, Brook RH (1998). How good is the quality of health care in the United States?. Milbank Q.

[38] Grol R (2001). Successes and failures in the implementation of evidence-based guidelines for clinical practice. Med Care.

[39] Shaneyfelt TM (2001). Building bridges to quality. JAMA.

[40] Guyatt GH, Meade MO, Jaeschke RZ, Cook DJ, Haynes RB (2000). Practitioners of evidence based care. Not all clinicians need to appraise evidence from scratch but all need some skills. BMJ.

[41] Grimshaw JM, Thomas RE, MacLennan G, Fraser C, Ramsay CR, Vale L (2004). Effectiveness and efficiency of guideline dissemination and implementation strategies. Health Technol Assess.

[42] OECD report (2012). Policies for a revitalisation of Japan.

[43] Kusuda S, Fujimura M, Uchiyama A, Totsu S, Matsunami K (2012). Trends in morbidity and mortality among very low birth weight infants from 2003 to 2008 in Japan. Pediatr Res.

[44] Itabashi K, Horiuchi T, Kusuda S, Kabe K, Itani Y, Nakamura T (2009). Mortality rates for extremely low birth weight infants born in Japan in 2005. Pediatrics.

[45] Davis DA, Taylor-Vaisey A (1997). Translating guidelines into practice. A systematic review of theoretic concepts, practical experience and research evidence in the adoption of clinical practice guidelines. CMAJ.

[46] Jewell D (2003). How to change clinical behaviour: no answers yet. Br J Gen Pract.

[47] Forsetlund L, Bjorndal A, Rashidian A, Jamtvedt G, O'Brien MA, Wolf F (2009). Continuing education meetings and workshops: effects on professional practice and health care outcomes. Cochrane Database Syst Rev.

