# Supplementary material for: The effect of professional-led guideline workshops on clinical practice for the management of patent ductus arteriosus in preterm neonates in Japan: a controlled before-and-after study
Source: Implement Sci. 2015 May 8;10:67. doi: 10.1186/s13012-015-0258-5 (PMC4438511; doi:10.1186/s13012-015-0258-5)
Supplement: Additional file 1: — Evidence- and consensus-based clinical practice guideline for treatment of patent ductus arteriosus (PDA) in preterm infants (English version). This file is an English translation of 33 recommendations in the ‘Evidence and Consensus Based Clinical Practice Guideline for Treatment of Patent Ductus Arteriosus of Preterm Infants’ that was originally published in Japanese. [file 13012_2015_258_MOESM1_ESM.pdf]

Evidence- and consensus-based clinical practice guideline for treatment of patent ductus arteriosus (PDA) in preterm infants

Developed by Japanese Preterm PDA (J-Prep) Guideline Development Team

This is an English version of the Japanese guideline recommendations published as follows:
Japanese Preterm PDA (J-Prep) Guideline Development Team. Konkyo to soui ni motozuku mijukuji doumyakukan kaizonshou chiryou gaidorain. [Evidence- and consensus-based clinical practice guideline for treatment of patent ductus arteriosus (PDA) in preterm infants]. *Journal of Japan Society for Premature and Newborn Medicine.* 2010;22(2):77-89 [in Japanese].

The guideline was developed according to the international standards for assessing the quality of practice guidelines (AGREE).[1]

Strength of evidence (Grading of recommendation)

Study design and quality	Studies with high quality and high applicability	Studies with moderate applicability requiring careful consideration	Studies with little applicability
Randomized controlled trials or systematic reviews of randomized controlled trials	1++	1+	1-
Non-randomized controlled trials or analytic epidemiological studies	2++	2+	2-
Case series, case reports, or expert opinions	3++	3+	3-

Grades for recommendations

1++	**Grade A**
1+, 2++, 2+	**Grade B**
3++, 3+	**Grade C**
Studies with negative strength of evidence (1-, 2-, 3-) are not used to make recommendations.	

The above grades indicate confidence or strength of evidence rather than importance.

Clinical questions (CQ) and Recommendations (Rec.)

CQ 1	What are the risk factors for patent ductus arteriosus (PDA) in preterm infants?	Grades
Rec. 1	Excessive fluid administration increases the risk of PDA in preterm infants and should be avoided for low birthweight infants.	B
Rec. 2	From the viewpoint of preventing PDA in preterm infants, surfactant administration for the treatment of respiratory distress syndrome is recommended.	A
Rec. 3	From the viewpoint of preventing PDA in preterm infants, routine administration of furosemide for infants with respiratory distress syndrome is not recommended.	C
Rec. 4	From the viewpoint of preventing PDA in preterm infants, antenatal corticosteroids administration to women who are likely to deliver infants at < 35 weeks gestation is recommended.	C
Rec. 5	There is little evidence to show how postnatal systemic corticosteroids administered to prevent chronic lung disease affect PDA in preterm infants. Given concerns about the risk of intestinal perforation and long-term neurological impact of postnatal corticosteroids, the use of corticosteroids should be based on careful consideration and balancing of the risks and benefits.	B
Rec. 6	There is little evidence that the following factors are risk factors for PDA in preterm infants:<br>• Delayed cord clamping, prophylactic surfactant administration, high frequency oscillatory ventilation, synchronized intermittent mechanical ventilation, volume-targeted ventilation, inhaled nitric oxide, continuous positive airway pressure support, permissive hypercapnia, inhaled corticosteroids, phototherapy, and administration of thyroid hormone, dopamine, intravenous lipid emulsions, albumin preparation, or morphine<br>These treatments need to be tailored to individual patients' diseases and conditions.	C
CQ 2	Is prophylactic (routine) administration of cyclooxygenase (COX) inhibitors more effective than selective administration to improve preterm infants' outcomes?	
Rec. 7	Prophylactic administration of indomethacin is recommended to prevent PDA in preterm infants. Each institution or NICU should decide the indication for the prophylaxis based on its capability to perform PDA ligation as well as the rates of symptomatic PDA and intraventricular hemorrhage for each category of gestational age or birth weight.	A
Rec. 8	Prophylactic ibuprofen is not recommended to prevent PDA in preterm infants.	A
CQ 3	Which COX inhibitors are more effective for early prophylactic use after birth to improve preterm infants' outcomes: indomethacin, mefenamic acid, ibuprofen, or slindac?<br>Also, what administration methods (doses, intervals, and routes of administration) are more effective to improve preterm infants' outcomes?	

Rec. 9	Among the COX inhibitors, indomethacin is recommended for prophylactic administration.	B
Rec. 10	For prophylactic use, intravenous indomethacin administration should commence within 6 hours after birth at 0.1mg/kg/dose over 6 hours.<br>If ductus arteriosus does not close, indomethacin can be administered every 24 hours up to three times as a prophylactic administration.	C
CQ 4	Not available.<br>(The CQ 4 was supposed to examine issues related to diagnosis of PDA; however, it was not included in this guideline.)	
CQ 5	Which treatment is the most effective for symptomatic PDA in preterm infants, conservative management (medical treatment other than COX inhibitors), administration of COX inhibitors, or surgical ligation to improve preterm infants' outcomes?	
Rec. 11	As an initial treatment of symptomatic PDA in preterm infants, administration of COX inhibitors is recommended rather than other medical treatments (e.g. fluid restriction, etc.).	B
Rec. 12	Routine PDA ligation is not recommended as an initial treatment of symptomatic PDA in preterm infants.	B
CQ 6	Which COX inhibitors are recommended to treat symptomatic PDA in preterm infants: indomethacin, mefenamic acid, ibuprofen, or slindac?<br>What administration methods (doses, intervals, and routes of administration) of COX inhibitors are recommended to treat symptomatic PDA?	
Rec. 13	Among COX inhibitors, indomethacin is recommended to treat symptomatic PDA in preterm infants.	A
Rec. 14	As a treatment of symptomatic PDA in preterm infants, indomethacin should be administered intravenously at 0.1 to 0.2 mg/kg/dose every 12 to 24 hours up to three times consecutively.	B
Rec. 15	Rapid intravenous injection of indomethacin is not recommended.	C
CQ 7	During a course of COX inhibitors in preterm infants, what clinical symptoms or laboratory findings should be checked or monitored?	
Rec. 16	During a course of COX inhibitors in preterm infants, at a minimum the following outputs and levels should be checked or monitored regardless of the total number of administrations:<br>• Urine outputs and serum creatinine levels during prophylactic use of indomethacin<br>• Urine outputs, serum creatinine levels, and serum glucose levels during PDA treatment with indomethacin	A<br>B
Rec. 17	During a course of COX inhibitors in preterm infants, the clinical and radiographic signs of necrotizing enterocolitis or intestinal perforation should be carefully monitored	C

	regardless of the total number of administrations:<br>• Clinical signs: abdominal distention, bloody stool, bile gastric aspirate, abdominal discoloration, etc.<br>• Radiographic signs: pneumatosis intestinalis, portal venous gas, pneumoperitoneum, etc.	
Rec. 18	In particular, the signs of necrotizing enterocolitis should be carefully monitored when indomethacin is given four times or more consecutively.	B
CQ 8	Is the discontinuation of enteral feeding more effective than the continuation of enteral feeding to prevent necrotizing enterocolitis or intestinal perforation during a course of COX inhibitors for PDA treatment in preterm infants?	
Rec. 19	Routine discontinuation of enteral feeding is not recommended during a course of COX inhibitors for PDA treatment in preterm infants.	C
CQ 9	Is adjustment of the fraction of inspired oxygen (FiO_2) more effective than no adjustment to treat PDA with COX inhibitors in preterm infants?	
Rec. 20	Routine adjustment of FiO_2 is not recommended to treat PDA with COX inhibitors in preterm infants. Increasing FiO_2 may contribute to a constriction of PDA. Decreasing FiO_2 may be considered when pulmonary blood flow increases with persistent PDA.	C
CQ 10	During a course of COX inhibitors for PDA treatment, is the restriction of fluid intake more effective to treat PDA in preterm infants than no restriction (liberal fluid management)?	
Rec. 21	During a course of COX inhibitors for PDA treatment, it is recommended to adjust fluid intake to avoid excessive intake while paying attention to signs of dehydration and circulatory failure to prevent excessive fluid restriction.	B
CQ 11	Is the administration of catecholamine (dopamine and dobutamine) effective to close PDA and improve renal function caused by indomethacin treatment for PDA in preterm infants?	
Rec. 22	Routine administration of dopamine to prevent or treat renal failure caused by indomethacin treatment for PDA in preterm infants is not recommended.	B
Rec. 23	Routine administration of dobutamine to prevent or treat renal failure caused by indomethacin treatment for PDA in preterm infants is not recommended.	C
Rec. 24	Routine administration of dopamine and dobutamine to treat PDA in preterm infants is not recommended.	C
CQ 12	Is routine red blood cell transfusion more effective than selective transfusion to treat PDA with indomethacin?	
Rec. 25	Routine red blood cell transfusion is not recommended during PDA treatment with indomethacin in preterm infants.	B
CQ 13	Is routine administration of steroids more effective than selective transfusion to treat PDA with indomethacin?	

Rec. 26	Routine administration of steroids is not recommended during PDA treatment with indomethacin in preterm infants.	C
CQ 14	Is routine administration of vitamin A more effective than selective transfusion to treat PDA with indomethacin?	
Rec. 27	Routine administration of vitamin A is not recommended during PDA treatment with indomethacin in preterm infants.	B
CQ 15	Is routine administration of furosemide more effective than selective transfusion to treat PDA with indomethacin?	
Rec. 28	Routine administration of furosemide is not recommended during PDA treatment with indomethacin in preterm infants.	B
Rec. 29	Furosemide should not be used during a course of indomethacin to treat PDA when the ratio of blood-urea-nitrogen (mg/dl) to creatinine (mg/dl) is 20 mg or more with signs of dehydration.	B
CQ 16	What criteria for PDA ligation (clinical or radiographic signs, laboratory tests, etc.) are most effective to be used to improve infants' outcomes?	
Rec. 30	The decision of surgical PDA ligation should be based on infants' circulatory and respiratory conditions, nutritional status, renal function, and radiographic signs (X-rays and/or echocardiography).<br>Also, it is recommended to consider the risks and benefits of continuing observation or medical treatments (fluid restriction, indomethacin treatment, etc.) without surgical ligation along with the experience and surgical outcomes of PDA ligation in each institution.	C
Rec. 31	It is recommended to decide surgical PDA ligation early, if preterm infants develop renal failure or necrotizing enterocolitis due to cardiac failure with PDA, balancing overall risks and benefits of surgical ligation in each institution.	C
CQ 17	Do high-volume institutions have less surgical complications for PDA ligation than low-volume institutions?	
Rec. 32	If possible, PDA ligation should be performed at high-volume institutions rather than low-volume institutions.	C
CQ 18	Which is the most effective treatment for indomethacin-resistant late-stage PDA to improve preterm infants' outcomes: observation, further repetition of indomethacin courses, or PDA ligation?	
Rec. 33	There is little evidence to show which treatment is effective for indomethacin-resistant late-stage PDA or re-opening late-stage PDA in preterm infants. It is recommended that late-stage PDA management is based on the following considerations:<br>• Decision of treatment should be based on the degree or severity of increased pulmonary blood flow, reduced systemic blood flow, and cardiac failure.<br>• Management of late-stage PDA includes: 1) observation, 2) continuing indomethacin treatment, and 3) PDA ligation.	C

	• Watchful observation is recommended for infants with late-stage PDA when they do not have any significant signs or symptoms of PDA such as respiratory problems due to increased pulmonary blood flow, heart failure requiring fluid restriction, renal failure (or oliguria) due to reduced systematic blood flow, etc.<br>• PDA ligation is recommended without delay for infants with late-stage PDA who require respiratory support due to increased pulmonary blood flow or fluid restriction for cardiac failure, who develop oliguria or renal failure due to reduced systemic blood flow, or who have complications due to COX inhibitors (e.g. intestinal problems, etc.).	

Reference:

1. **Development and validation of an international appraisal instrument for assessing the quality of clinical practice guidelines: the AGREE project.** *Qual Saf Health Care* 2003, **12:**18-23.
