# Supplementary material for: The effect of professional-led guideline workshops on clinical practice for the management of patent ductus arteriosus in preterm neonates in Japan: a controlled before-and-after study
Source: Implement Sci. 2015 May 8;10:67. doi: 10.1186/s13012-015-0258-5 (PMC4438511; doi:10.1186/s13012-015-0258-5)
Supplement: Additional file 2: — Evaluation of physicians’ confidence for PDA diagnosis, prophylaxis, and treatment management. This file is a questionnaire on physicians’ confidence for PDA diagnosis, prophylaxis, and treatment. [file 13012_2015_258_MOESM2_ESM.docx]

**Additional file 1: Evaluation of physicians’ confidence for PDA diagnosis, prophylaxis and treatment management**

| **Evaluation of confidence in PDA management** | **Confidence rating** |
| --- | --- |
| Prophylactic administration of indomethacin |  |
| Treatment of symptomatic PDA |  |
| Judgement of indication for surgical treatment of symptomatic PDA |  |
| Monitoring of clinical conditions and adverse events and assessment of laboratory results during PDA treatment with indomethacin |  |
| Additional treatment of PDA (transfusion, diuretics, catecholamine, corticosteroids, vitamin A, etc.) |  |
| Management of PDA in chronic phase |  |
| Fluid management during PDA treatment |  |
| Respiratory management during PDA treatment |  |
| Nutrition management during PDA treatment |  |

Confidence was rated as CR = 1: Not confident; CR = 2: Satisfactory, but lacking confidence; CR = 3: Confident in some cases, but would like more experience; CR = 4: Fully confident in most cases.
