# Supplementary material for: The effect of professional-led guideline workshops on clinical practice for the management of patent ductus arteriosus in preterm neonates in Japan: a controlled before-and-after study
Source: Implement Sci. 2015 May 8;10:67. doi: 10.1186/s13012-015-0258-5 (PMC4438511; doi:10.1186/s13012-015-0258-5)
Supplement: Additional file 3: — Site investigators of the Neonatal Research Network of Japan. This file is a full list of the names of site investigators of the Neonatal Research Networks of Japan. [file 13012_2015_258_MOESM3_ESM.docx]

**Additional file 3. Site investigators of the Neonatal Research Network of Japan**

Fujimura M (Director, Neonatal Research Network of Japan); Kusuda S (Associate Director, Neonatal Research Network of Japan); Hattori S (Sapporo City General Hospital); Noro A (Kushiro Red Cross Hospital); Amizuka T (Aomori Prefectural Central Hospital); Chida S (Iwate Medical University); Takahashi R (Sendai Red Cross Hospital); Arai H (Akita Red Cross Hospital); Imamura T (Fukushima Medical University); Ujiie N (National Fukushima Hospital); Miyazono Y (University of Tsukuba); Shimizu J (Tsuchiura Kyodo General Hospital); Suzumura H (Dokkyo Medical University); Kono Y( Jichi Medical University); Shimizu M (Saitama Children's Medical Center); Kunikata T (Saitama Medical University Saitama Medical Center); Fujiu T (Gunma Children's Medical Center); Sato H (Kameda Medical Center); Kondo T (Tokyo Women’s Medical University Yachiyo Medical Center); Watanabe T (Tokyo Metropolitan Bokuto Hospital); Aizawa M (Showa University); Uchiyama A (Tokyo Women’s Medical University); Makimoto M (Nihon University Itabashi Hospital); Hoshi J (Teikyo University); Yoda H (Toho University); Kawakami Y (Japan Red Cross Medical Center); Ishii N (Aiiku Hospital); Ito Y (National Center for Child Health and Development); Itani H (Kanagawa Children's Medical Center); Seki K (Yokohama City University Medical Center); Nomura M (Tokai University); Nowatari M (Kitazato University); Nemoto A (Yamanashi Prefectural Central Hospital); Nagata O (Nagaoka Red Cross Hospital); Nagayama Y (Niigata City Hospital); Nakamura T (Nagano Children’s Hospital); Okada M (Shinshu University); Nakata S (Iida City Hospital): Shimazaki E (National Nagano Hospital); Yoda T (Saku General Hospital); Hutatani T (Toyama Prefectural Central Hospital); Ueno Y (Ishikawa Prefectural Central Hospital); Iwai K (Fukui Prefectural Hospital); Nakazawa Y (Shizuoka Children’s Hospital); Oki S (Seirei Hamamatsu General Hospital); Suzuki C (Nagoya Red Cross First Hospital); Bonno M (National Mie Hospital); Kawano Y (Gifu Prefectural Central Hospital): Nakamura K (Otsu Red Cross Hospital); Mitsufuji N (Kyoto Red Cross First Hospital); Shiraishi J (Osaka Medical Center and Research Institute for Maternal and Child Health); Ichiba H (Osaka City General Hospital); Minami H (Takatsuki Hospital); Wada H (Yodogawa Christian Hospital); Ohashi A (Kansai Medical University); Sumi K (Aizenbashi Hospital); Takahashi Y (Nara Medical University); Okutani T (Wakayama Prefectural Medical University); Yoshimoto S (Hyogo Prefectural Kobe Children's Hospital); Nagata I (Tottori University); Kato E (Shimane Prefectural Central Hospital); Watabe S (Kurashiki Central Hospital); Kageyama M (National Okayama Hospital); Fukuhara R (Hiroshima Prefectural Hospital); Hayashitani M (Hiroshima City Hospital); Hasegawa K (Yamaguchi Prefectural Medical Center); Ohta A (National Kagawa Children's Hospital); Kuboi T (Kagawa University); Akiyoshi S (Ehime Prefectural Central Hospital); Kikkawa K (Kochi Health Sciences Center); Saijo T (Tokushima University); Shimokawa S (St. Mary's Hospital); Matsumoto N (Kitakyushu City Municipal Medical Center); Kanda H (Kurume University); Oota E (Fukuoka University); Kanda G (National Kyushu Medical Center); Ochiai M (Kyushu University); Aoki M (National Nagasaki Medical Center); Kondo Y (Kumamoto City Hospital); Iwai M (Kumamoto University); Iida K (Oita Prefectural Hospital); Ikenoue T (Miyazaki University); Ibara S (Kagoshima City Hospital); Kohama M (Okinawa Chubu Hospital).
